# *Serratia marcescens* Infections in Neonatal Intensive Care Units (NICUs)

**DOI:** 10.3390/ijerph16040610

**Published:** 2019-02-20

**Authors:** Maria Luisa Cristina, Marina Sartini, Anna Maria Spagnolo

**Affiliations:** Department of Health Sciences, University of Genoa, Italy Via Pastore, 1-16132 Genoa, Italy; cristinaml@unige.it (M.L.C.); sartini@unige.it (M.S.)

**Keywords:** *Serratia marcescens*, outbreaks, NICUs

## Abstract

*Serratia marcescens* belongs to the family Enterobacteriaceae, which is commonly found in water, soil, animals, insects, plants. Although *S. marcescens* displays relatively low virulence, it causes nosocomial infections and outbreaks in severely immunocompromised or critically ill patients, particularly in settings such as intensive care units (ICUs), especially neonatal units (NICUs). This microorganism gives rise to a wide range of clinical manifestations in newborns: from asymptomatic colonization to keratitis, conjunctivitis, urinary tract infections, pneumonia, surgical wound infections, sepsis, bloodstream infection and meningitis. The most frequent site of infection is the bloodstream, followed by the respiratory apparatus and the gastrointestinal tract. Strains of *S. marcescens* involved in epidemic events have frequently proved to be multi-resistant. Indeed, this species displays intrinsic resistance to several classes of antibiotics. Often, the specific source of the infection cannot be identified. However, the contaminated hands of healthcare workers are believed to be a major vehicle of its transmission. In neonatal intensive care units, colonized or infected newborns are the main potential source of *S. marcescens*, particularly in the respiratory apparatus, but also in the gastrointestinal tract. The early identification of colonized or infected patients and the prompt implementation of infection control measures, particularly rigorous hand hygiene and contact precautions, are essential in order to curb the spread of infection.

## 1. Introduction

The hospital environment can play an essential role in the transmission of multidrug resistant pathogens, [1] including *Serratia marcescens* which is able to survive in most of them.

Until late in the 20th century, *S. marcescens* was considered a nonpathogenic saprophytic organism. Its pathogenicity in humans was first noted in 1913; however, the prevalence of *S. marcescens* in human diseases was underestimated for years, until the first known outbreak of nosocomial *S. marcescens* infection in 1951. Since then, infections due to this organism have been reported increasingly frequently [2,3].

Although *S. marcescens* displays relatively low virulence, it causes nosocomial infections in severely immunocompromised or critically ill patients, particularly in settings such as intensive care units (ICUs), especially neonatal units (NICUs) [4,5].

*S. marcescens* gives rise to a wide range of clinical manifestations in newborns: from asymptomatic colonization to keratitis, conjunctivitis, urinary tract infections, pneumonia, surgical wound infections, sepsis, bloodstream infection and meningitis [6,7]. The most frequent site of infection, however, is the bloodstream, followed by the respiratory apparatus and the gastrointestinal tract [8].

The aim of the review is to summarize data on characteristics of the *Serratia marcescens*, antibiotic resistance, epidemiology, risk factors, sources of infections and prevention and control measures.

### Characteristics of the Microorganism and Antibiotic Resistance

The genus *Serratia* belongs to the family Enterobacteriaceae and comprises at least 14 species and two subspecies. Of these *Serratia* species, *S. marcescens* is the one most commonly associated with human infections [9].

This microorganism is a mobile bacterium that is able to grow at temperatures between 5 °C and 40 °C and in media with pH values between 5 and 9. Common habitats of *S. marcescens* are water, soil, animals, insects, plants [7].

The name “marcescens” derives from the rapid transformation into a viscous fluid mass following the production of an intense red pigment (prodigiosin). *S. marcescens* is a heterogeneous species and probably contains an unlimited number of different clones. A study by Casolari et al. found a marked difference in pathogenicity between two strains of *S. marcescens* and a proportionally inverse relationship between virulence and the amount of pigment (prodigiosin) produced [10].

Many other aspects of the pathogenicity and virulence of *S. marcescens* have been studied, including adherence and hydrophobicity, and lipopolysaccharide (LPS) and extracellular products. Two modes of adhesion to host epithelial surfaces have been suggested. These are mannose-resistant (MR) pili and mannose-sensitive (MS) pili. LPS, which is responsible for the biological activity of endotoxin, has been investigated fully and 24 somatic antigens have been described. The production of different enzymes by *S. marcescens* as virulence factors has also been reported, including chitinase, lipase, chloroperoxidase and an extracellular protein, HasA [9,11].

Strains of *S. marcescens* involved in epidemic events have frequently proved to be multi-resistant. Indeed, this species displays intrinsic resistance to several classes of antibiotics, including some β-lactams and tetracyclines. *S. marcescens* is intrinsically susceptible to other classes of antimicrobials, including quinolones and aminoglycosides, though chromosomal or plasmid-mediated resistance to some of these has been reported [12,13,14]. Data on the whole genome sequencing of *S. marcescens* isolated from environmental and clinical samples have only recently become available [9,15,16,17]. Taken together, these data indicate that the genome of *S. marcescens* is highly dynamic, which reflects the diverse environmental niches that the bacterium occupies and its nature as an opportunistic pathogen.

Many of the clinical isolates of this organism carry chromosomal and plasmid-encoded genetic determinants that confer resistance to a wide range of antibiotics, including extended-spectrum β-lactamase (ESBL) or metallo β-lactamase (MBL) [9].

In a study conducted in two Polish hospitals in the period 1996–2000, 19% (67/354) of *S. marcescens* isolates were seen to produce ESBL [18]. Similarly, a 2-year investigation carried out in Taiwan revealed that 12% (15/123) of isolates were ESBL+ and that the 30-day mortality rate in patients with infections due to these strains was 33% [19].

MBL-producing *S. marcescens* strains are clinically more problematic, as they are highly resistant to a broader range of β-lactams. A representative MBL enzyme, IMP-1, was first observed in a clinical isolate of *S. marcescens* in 1991 in Japan [20]. Since then, various types of MBL have been identified in many strains of *S. marcescens*, including those responsible for epidemics [9].

A positive correlation between resistance to antibiotics and to some biocidal agents (i.e., cetrimide and chlorhexidine) was described in 1991 for *Serratia marcescens* [21].

*Serratia marcescens*, have exhibited high intrinsic resistance and survival after exposure to quaternary ammonium compounds [22].

## 2. Epidemiology

In the literature, there has been a very large number of reported hospital-related *S. marcescens* outbreaks.

According to Gastmeier and Raymond, *Serratia* spp. is the third most frequent pathogen involved in outbreaks in neonatal facilities [8] and accounts for 15% of all isolates from nosocomial infections in such settings [23].

In Europe, the most recent data from the *HAI-Net* of the European Centre for Disease Prevention and Control (ECDC) indicate that in 2016 *Serratia* spp. was the sixth most frequently isolated microorganism in patients with pneumonia hospitalized in European ICUs (5.2 per 100 microorganisms), and was in 10th and 9th place, respectively, in those with bloodstream infections (3.3 per 100 microorganisms) and urinary tract infections (1.6 per 100 microorganisms) [6,24].

Casolari et al. investigated two consecutive *S. marcescens* outbreaks which occurred in a NICU of a tertiary level hospital in North Italy in a period of 10 years*. S. marcescens* occurred in 127 neonates: 43 developed infection and 3 died. Clinical infections had different severity and localization. The most severe pictures were sepsis (21%) and pneumonia (19%), related to the three fatal cases. The overall mortality was 7%. Seven clusters were recorded due to 12 unrelated clones which persisted for years in the ward, although no environmental source was found. The main epidemic clone A sustaining the first cluster reappeared in seven years later as an extended spectrum β-lactamase (ESBL)-producing strain and supporting the second epidemic. The opening of a new ward for non-intensive care-requiring neonates, strict adherence to alcoholic hand disinfection, the timely identification and isolation of infected and colonized neonates assisted in containing the epidemics. Genotyping was effective in tracing the evolution and dynamics of the clones demonstrating their long-term persistence in the ward [10].

Arslan et al. described an outbreak of sepsis caused by *S. marcescens* in a NICU that involved seven patients, following the administration of parenteral nutrition (PN). One of the newborns was premature and died. Authors identified the source of the outbreak using RAPD-PCR and plasmid profile analysis as well as PFGE.

The results strongly suggest that the nosocomial sepsis by *S. marcescens* in the newborns was caused by the administration of the contaminated PN.

Investigation of the practice of care identified several factors that were not in accordance with general infection control standards. These included inadequate surface disinfection through incorrectly calculated disinfection solutions and inadequate hand hygiene practices of some staff members [25].

Redondo-Bravo et al. described an outbreak affecting a total of 59 neonates. Twenty-five (42.37%) neonates sustained an infection, most frequently conjunctivitis and sepsis. Environmental samples and samples from health care workers (HCWs) were obtained for microbiological analysis. The genetic relationships between the isolates were determined by automated repetitive sequence based polymerase chain reaction. Among all environmental samples, only three were positive for *S. marcescens*, all of which were obtained from siphons located in different pods. Of these, only one strain seemed to be related to the outbreak according to the molecular typing results [26].

In a study published by Madani et al. was described an outbreak involving 11 babies in a NICU and three babies in a nursery, infected with *S. marcescens* at a University Hospital in Saudi Arabia. 

Overall, fifteen infections were identified among 11 newborns in the NICU: septicaemia (five cases), purulent conjunctivitis (three), urinary tract infection (two), meningitis (two) and cellulitis (one). 

Three newborns in the nursery had three infections: purulent conjunctivitis (two cases) and omphalitis (one). Thirteen of 14 babies recovered fully but one died from *S. marcescens* meningitis and septicaemia. 

All infections were traced to intrinsically contaminated baby shampoo introduced to the units five days before the first reported case. The outbreak, lasting two months, terminated following withdrawal of the shampoo product [27].

In recent years, outbreaks associated with extended-spectrum β-lactamase-producing or imipenem-resistant *S. marcescens* strains have emerged as a major infection control problem [28]. Such outbreaks typically spread rapidly, with potentially devastating consequences [4]. 

Rates of morbidity and mortality due to *S. marcescens* infections can vary markedly [29]. Indeed, the scientific literature reports mortality rates from 0 to 45% [30,31,32].

## 3. Risk Factors

In newborns, the main risk factors for the acquisition of *Serratia* infection are: immaturity of the immune system and low birth weight (<1500 g) in preterm newborns [8], length of stay (LOS) [33], and the use of antibiotics, especially if they are broad-spectrum and administered empirically [34]. In this regard, a study by Villari et al. suggested that empirical antibiotic therapy with ampicillin associated to an aminoglycoside (gentamicin or netilmicin) was a predisposing factor for the acquisition of *S. marcescens* in newborns involved in an epidemic event [35]. 

Regarding LOS, Friedman found that LOS in newborn services unit >30 days (OR,4.4; 95% CI: 1.8–10.6; *p* =0.001) was independent risk factor for acquisition of *S marcescens* [33].

In a case-control study published by Al Jarousha et al., *S. marcescens* was detected in blood cultures of 159 confirmed cases of nosocomial bloodstream infections (BSIs). Seventy (44%) of these neonates died from *S. marcescens* infection and 89 recovered. This study compared patients with *S. marcescens* sepsis with uninfected controls. On multivariate analysis, risk factors significantly associated with *S. marcescens* infection were: birth weight < 1500 g (OR,1.7; *p* = 0.026); <37 weeks gestational age (OR,2.0; *p* = 0.002); and the use of mechanical ventilation (OR,2.3; *p* = 0.001) [2,36].

Moreover, in an outbreak involving 10 newborns in a NICU, Assadian et al. found that antibiotic treatment with cefuroxime, administered for nine days to the mother of the index case, was a possible risk factor for infection. No microbiological culture from the mother proved positive for *S. marcescens*. However, it is possible that the newborn had been initially colonized through close contact with the treated mother, given that cefuroxime (a second-generation cephalosporin) is known to be able to select *S. marcescens* in the intestine [37].

The main risk factor for the development of sepsis and bacteremia due to *Serratia* spp. is hospitalization. 

Another risk factor is exposure to invasive maneuvers, such as the placement of venous, intraperitoneal or urinary catheters and of mechanical ventilation devices [38].

Indeed, a frequent observation is that patients with urinary tract infections have often undergone recent surgery or the insertion of devices into the urinary tract. Further risk factors include urinary tract obstruction and renal insufficiency. 

With regard to infections of the respiratory apparatus, the use of mechanical ventilators is considered to be a risk factor. Moreover, in premature babies and newborns, previous sepsis can facilitate the development of meningitis or cerebral abscesses. 

## 4. Sources of Infections

Often, the specific source of the infection cannot be identified. However, the contaminated hands of healthcare workers are believed to be a major vehicle of its transmission. According to WHO adherence of HCWs to recommended hand hygiene procedures has been reported with very variable figures, in some cases unacceptably poor, with mean baseline rates ranging from 5% to 89%, representing an overall average of 38.7% [39].

In a study by Jang et al. cross-transmission through the transitory contamination of hands was seen to be the main cause of the spread of the infection. In their study, during an examination that had been agreed upon with the medical and nursing staff of a NICU, 24 hand-washes were carried out, three of which proved positive for *S. marcescens*. When analyzed by means of Pulsed-field gel electrophoresis (PFGE), the strains isolated from the hand-washes and those detected in three samples taken from the door of an incubator displayed the same pattern as the strain involved in the clinical outbreak [32].

High patient density and low nurse-to-patient ratio have also been reported as potential sources for the spread of the pathogen via contaminated hands of healthcare workers [10].

In neonatal intensive care units, colonized or infected newborns are the main potential source of *S. marcescens*, particularly in the respiratory apparatus, but also in the gastrointestinal tract [40]. 

In fact, *S. marcescens* is frequently detected in the feces of newborns in NICUs, even though this microorganism is not a normal component of the early intestinal microbial population in healthy newborns.

However, once their intestine is colonized by *S. marcescens* from outside, these newborns may become the source of epidemic foci. This makes it very difficult to control epidemics and underlines the importance of hand hygiene, since the hands of healthcare staff are probably the main vehicle of transmission of the pathogen. 

Once the microorganism has been acquired, many newborns remain colonized for long periods, especially at the gastrointestinal level, despite antibiotic treatment [41], though the modality of acquisition of the infection often remains unknown [10].

The reservoirs most frequently associated with outbreaks of nosocomial infection, particularly in neonatal intensive care units are: washbasins [30], tap water, air-conditioning systems [4], bronchoscopes, laryngoscopes [42], nebulizers, ventilation equipment, milk-drawers, mother’s milk [5], injectable solutions, liquid soap dispensers [43,44], baby shampoo [27], contaminated disinfectants [35,37,45], contaminated propofol [46], contaminated parenteral nutrition [25], prefilled syringes containing heparin or normal saline [47], intravenous magnesium sulphate [48], injection of compounded betamethasone [49], total parenteral nutrition contaminated with insulin in multi-dose vials [50], multi-dose vials of heparinized-saline [51], extrinsic contamination of the parenteral narcotic fentanyl by a healthcare worker (HCW) [52], neonatal incubator [32].

## 5. Prevention and Control Measures

As is well known, the strategies to be implemented in order to prevent hospital infections effectively are based on the awareness of possible reservoirs, the modes of transmission and the epidemiology of the microorganisms involved [53].

*S. marcescens* is a ubiquitous pathogen, the complete eradication of which from the environment is often difficult [41]; this means that it is also difficult to control epidemics caused by this microorganism, which sometimes persists for months or even years [28].

The early diagnosis of patients who are colonized or infected and the rapid implementation of infection control measures are key factors in curbing the spread of *S. mercescens* [10].

Ongoing surveillance is an integral part of infection control programs and enables prompt identification of outbreaks that are not directly evident, thereby allowing efficacious control interventions to be undertaken [6]. In this regard, a study conducted by Gastmeier et al. on several outbreaks in NICUs has shown that surveillance cultures of samples taken from patients, together with such measures as hand hygiene and the isolation or cohorting of infected newborns, have been introduced as routine procedures in order to control and interrupt epidemics in progress [8].

The recommendations of the CDC’s Healthcare Infection Control Practices Advisory Committee (HICPAC) emphasize the importance of a collaborative multidisciplinary approach to controlling hospital infections. Such interventions include staff training, environmental sampling, the isolation of colonized patients, the implementation of infection control measures, and awareness of the role of cross-contamination [4,54,55,56,57].

Several studies have evaluated the efficacy of possible strategies for reducing healthcare-related infections in NICUs. In this regard, such measures as limiting antibiotic therapy, reducing the use of invasive procedures, and drawing up guidelines on hygiene—to be implemented through training programs—have proved particularly efficacious. In cases in which the introduction of standard measures of hygiene have proved insufficient to arrest the outbreak, hospitals have even resorted to closing the units involved [2,58]. 

However, the efficacy of this solution has not been demonstrated, and is neither routinely suggested nor actuated [8].

The control measures to adopt should therefore include: (a) reviewing all healthcare procedures that may constitute a potential risk of infection; (b) training staff in proper and rigorous hand-washing and the use of gloves; (c) utilizing alcohol-based antiseptic gel; (d) adopting contact precautions; (e) improving the cleansing and disinfection of equipment and the environmental surfaces; they constitute a possible transitory site for the accumulation of microorganisms, which may be deposited on them through contact with the hands of healthcare personnel and patients or with infected instruments and materials [59,60,61].

In addition, in the event of suspected outbreaks are considered advisable: 1) the use of dedicated medical devices (e.g., stethoscope) and of dedicated personnel; 2) cohorting colonized and infected newborns; 3) active surveillance of all newborns, not only those of low birth weight, through periodic screening cultures; 4) temporarily limiting access to the unit; 5) transferring newborns as soon as possible. It is equally important that the implementation of these control measures be verified [5].

An extensive investigation of a nosocomial *S. marcescens* outbreak by van Ogtrop et al. highlighted the importance of wearing gloves when handling colonized or infected infants, since frequent hand carriage of *S. marcescens* was found when staff did not use gloves [62]. 

Indeed, 42% of hand cultures from healthcare workers were positive for *S. marcescens* after a working shift without using gloves, versus 3% when gloves were routinely used [40,46].

With regard to early screening, it has been seen to be useful to extend this to all newborns, not only those of low birth weight (<1500 g) as recommended by the German Commission for Hospital Hygiene and Infectious Diseases Prevention (KRINKO). Indeed, although the various epidemics of *S. marcescens* infection reported in NICUs in recent years have frequently involved preterm babies and those with low birth weight, in a recent German article reporting on a screening program involving all newborns, the majority of those colonized by *S. marcescens* were actually newborns with a birth weight above 1500 g [63].

In summary, the early identification of colonized or infected patients and the prompt implementation of infection control measures, particularly rigorous hand hygiene and contact precautions, are essential in order to curb the spread of infection.

## References

[1] Ottria G., Dallera M., Aresu O., Manniello M.A., Parodi B., Spagnolo A.M., Cristina M.L. (2010). Environmental monitoring programme in the cell therapy facility of a research centre: Preliminary investigation. J. Prev. Med. Hyg..

[2] Al Jarousha A.M., El Qouqa A., El Jadba A.H., Al Afifi A.S. (2008). An outbreak of *Serratia marcescens* septicaemia in neonatal intensive care unit in Gaza City, Palestine. J. Hosp. Infect..

[3] Su L.H., Ou J.T., Leu H.S., Chiang P.C., Chiu Y.P., Chia J.H., Kuo A.J., Chiu C.H., Chu C., Wu T.L. (2003). Extended epidemic of nosocomial urinary tract infections caused by *Serratia marcescens*. J. Clin. Microbiol..

[4] Uduman S.A., Farrukhy A.S., Nathz K.N.R., Zuhair M.Y., Ifrah A., Khawla A.D., Sunita P. (2002). An outbreak of *Serratia marcescens* infection in a special-care baby unit of a community hospital in United Arab Emirates: The importance of the air conditioner duct as a nosocomial reservoir. J. Hosp. Infect..

[5] Dessì A., Puddu M., Testa M., Marcialis M.A., Pintus M.C., Fanos V. (2009). *Serratia marcescens* infections and outbreaks in neonatal intensive care units. J. Chemother..

[6] Societa’ Italiana di Igiene, Medicina Preventiva e Sanita’ Pubblica (SItI)—Gruppo Italiano Studio Igiene Ospedaliera (GISIO). http://www.sitinazionale.org/bdsdocs/gisio/ricerca/01serratia.pdf.

[7] Mahlen S.D. (2011). Serratia infections: From military experiments to current practice. Clin. Microbiol. Rev..

[8] Gastmeier P., Loui A., Stamm-Balderjahn S., Hansen S., Zuschneid I., Sohr D., Behnke M., Obladen M., Vonberg R.P., Rüden H. (2007). Outbreaks in neonatal intensive care units-they are not like others. Am. J. Infect. Control.

[9] Iguchi A., Nagaya Y., Pradel E., Ooka T., Ogura Y., Katsura K., Kurokawa K., Oshima K., Hattori M., Parkhill J. (2014). Genome evolution and plasticity of *Serratia marcescens*, an important multidrug-resistant nosocomial pathogen. Genome Biol. Evol..

[10] Casolari C., Pecorari M., Fabio G., Cattani S., Venturelli C., Piccinini L., Tamassia M.G., Gennari W., Sabbatini A.M., Leporati G. (2005). A simultaneous outbreak of *Serratia marcescens* and *Klebsiella pneumoniae* in a neonatal intensive care unit. J. Hosp. Infect..

[11] Hejazi A., Falkiner F.R. (1997). *Serratia marcescens*. J. Med. Microbiol..

[12] Moradigaravand D., Boinett C.J., Martin V., Peacock S.J., Parkhill J. (2016). Recent independent emergence of multiple multidrug-resistant *Serratia marcescens* clones within the United Kingdom and Ireland. Genome Res..

[13] Livermore D.M., Winstanley T.G., Shannon K.P. (2001). Interpretative reading: Recognizing the unusual and inferring resistance mechanisms from resistance phenotypes. J. Antimicrob. Chemother..

[14] Stock I., Grueger T., Wiedemann B. (2003). Natural antibiotic susceptibility of strains of *Serratia marcescens* and the S. liquefaciens complex: S. liquefaciens sensu stricto, *S. proteamaculans* and *S. grimesii*. Int. J. Antimicrob. Agents.

[15] Chung W.C., Chen L.L., Lo W.S., Kuo P.A., Tu J., Kuo C.H. (2013). Complete genome sequence of *Serratia marcescens* WW4. Genome Announc..

[16] Liu P.Y., Huang Y.T., Lin S.Y., Chang G.C., Chen J.W. (2013). Draft genome sequence of the *Serratia marcescens* strain VGH107, a Taiwanese clinical isolate. Genome Announc..

[17] Wan Y., Gorrie C.L., Jenney A., Mirceta M., Holt K.E. (2015). Draft genome sequence of a clinical isolate of *Serratia marcescens*, strain AH0650_Sm1. Genome.

[18] Naumiuk L., Baraniak A., Gniadkowski M., Krawczyk B., Rybak B., Sadowy E., Samet A., Kur J. (2004). Molecular epidemiology of *Serratia marcescens* in two hospitals in Gdansk, Poland, over a 5-year period. J. Clin. Microbiol..

[19] Cheng K.C., Chuang Y.C., Wu L.T., Huang G.C., Yu W.L. (2006). Clinical experiences of the infections caused by extended-spectrum beta-lactamase-producing *Serratia marcescens* at a medical center in Taiwan. Jpn. J. Infect. Dis..

[20] Osano E., Arakawa Y., Wacharotayankun R., Ohta M., Horii T., Ito H., Yoshimura F., Kato N. (1994). Molecular characterization of an enterobacterial metallo beta-lactamase found in a clinical isolate of *Serratia marcescens* that shows imipenem resistance. Antimicrob. Agents Chemother..

[21] Kampf G. (2018). Biocidal agents used for disinfection can enhance antibiotic resistance in Gram-Negative species. Antibiotics.

[22] Buffet-Bataillon S., Tattevin P., Bonnaure-Mallet M., Jolivet-Gougeon A. (2012). Emergence of resistance to antibacterial agents: The role of quaternary ammonium compounds—A critical review. Int. J. Antimicrob. Agents.

[23] Raymond J., Aujard Y. (2000). Nosocomial infections in pediatric patients: A European, multicenter prospective study. European study group. Infect. Control Hosp. Epidemiol..

[24] European Centre for Disease Prevention and Control (2018). Healthcare-associated infections acquired in intensive care units. ECDC.

[25] Arslan U., Erayman I., Kirdar S., Yuksekkaya S., Cimen O., Tuncer I., Bozdogan B. (2010). *Serratia marcescens* sepsis outbreak in a neonatal intensive care unit. Pediatr. Int..

[26] Redondo-Bravo L., Gutiérrez-González E., San Juan-Sanz I., Fernández-Jiménez I., Ruiz-Carrascoso G., Gallego-Lombardo S., Sánchez-García L., Elorza-Fernández D., Pellicer-Martínez A., Omeñaca F. (2018). *Serratia marcescens* outbreak in a neonatology unit of a Spanish tertiary hospital: Risk factors and control measures. Am. J. Infect. Control.

[27] Madani T.A., Alsaedi S., James L., Eldeek B.S., Jiman-Fatani A.A., Alawi M.M., Marwan D., Cudal M., Macapagal M., Bahlas R. (2011). *Serratia marcescens*-contaminated baby shampoo causing an outbreak among newborns at King Abdulaziz University Hospital, Jeddah, Saudi Arabia. J. Hosp. Infect..

[28] Liou B.H., Duh R.W., Lin Y.T., Lauderdale T.L., Fung C.P. (2014). Taiwan Surveillance of Antimicrobial Resistance (TSAR) Hospitals. A multicenter surveillance of antimicrobial resistance in *Serratia marcescens* in Taiwan. J. Microbiol. Immunol. Infect..

[29] Fleisch F., Zimmermann-Baer U., Zbinden R., Bischoff G., Arlettaz R., Waldvogel K., Nadal D., Ruef C. (2002). Three consecutive outbreaks of *Serratia marcescens* in a neonatal intensive care unit. Clin. Infect. Dis..

[30] Sarvikivi E., Lyytikainen Ö., Salmenlinna S., Vuopio-Varkila J., Luukkainen P., Tarkka E., Saxén H. (2004). Clustering of *Serratia marcescens* infections in a neonatal intensive care unit. Infect. Control Hosp. Epidemiol..

[31] Messerschmidt A., Prayer D., Olischar M., Poliak A., Bimbacher R. (2004). Brain abscesses after *Serratia marcescens* infection on a neonatal intensive care unit: Differences on serial imaging. Neuroradiology.

[32] Jang T.N., Fung C.P., Yang T.L., Shen S.H., Huang C.S., Lee S.H. (2001). Use of pulsed-field gel electrophoresis to investigate an outbreak of *Serratia marcescens* infection in a neonatal intensive care unit. J. Hosp. Infect..

[33] Friedman N.D., Kotsanas D., Brett J., Billah B., Korman T.M. (2008). Investigation of an outbreak of *Serratia marcescens* in a neonatal unit via a case-control study and molecular typing. Am. J. Infect. Control.

[34] David M.D., Weller T.M., Lambert P., Fraise A.P. (2006). An outbreak of *Serratia marcescens* on the neonatal unit: A tale of two clones. J. Hosp. Infect..

[35] Villari P., Crispino M., Salvadori A., Scarcella A. (2001). Molecular epidemiology of an outbreak of *Serratia marcescens* in a neonatal intensive care unit. Infect. Control Hosp. Epidemiol..

[36] Voelz A., Müller A., Gillen J., Le C., Dresbach T., Engelhart S., Exner M., Bates C.J., Simon A. (2010). Outbreaks of *Serratia marcescens* in neonatal and pediatric intensive care units: Clinical aspects, risk factors and management. Int. J. Hyg. Environ. Health.

[37] Assadian O., Berger A., Aspöck C., Mustafa S., Kohlhauser C., Hirschl A.M. (2002). Nosocomial outbreak of *Serratia marcescens* in a neonatal intensive care unit. Infect. Control Hosp. Epidemiol..

[38] Buttinelli E., Ardoino I., Domeniconi G., Lanzoni M., Pugni L., Ronchi A., Mosca F., Biganzoli E., Castaldi S. (2017). Epidemiology of *Serratia marcescens* infections in NICU of a teaching and research hospital in northern Italy. Minerva Pediatr..

[39] World Health Organization (2009). WHO Guidelines on Hand Hygiene in Health Care: First Global Patient Safety Challenge Clean Care is Safer Care.

[40] Giles M., Harwood H.M., Gosling D.A., Hennessy D., Pearce C.T., Daley A.J. (2006). What is the best screening method to detect *Serratia marcescens* colonization during an outbreak in a neonatal intensive care nursery?. J. Hosp. Infect..

[41] Montagnani C., Cocchi P., Lega L., Campana S., Biermann K.P., Braggion C., Pecile P., Chiappini E., De Martino M., Galli L. (2015). *Serratia marcescens* outbreak in a neonatal intensive care unit: Crucial role of implementing hand hygiene among external consultants. BMC Infect. Dis..

[42] Cullen M.M., Trail A., Robinson M., Keaney M., Chadwick P.R. (2005). *Serratia marcescens* outbreak in a neonatal intensive care unit prompting review of decontamination of laryngoscopes. J. Hosp. Infect..

[43] Zapka C.A., Campbell E.J., Maxwell S.L., Gerba C.P., Dolan M.J., Arbogast J.W., Macinga D.R. (2011). Bacterial hand contamination and transfer after use of contaminated bulk-soap-refillable dispensers. Appl. Environ. Microbiol..

[44] Buffet-Bataillon S., Rabier V., Bétrémieux P., Beuchée A., Bauer M., Pladys P., Le Gall E., Cormier M., Jolivet-Gougeon A. (2009). Outbreak of *Serratia marcescens* in a neonatal intensive care unit: Contaminated unmedicated liquid soap and risk factors. J. Hosp. Infect..

[45] Jones B.L., Gorman L.J., Simpson J., Curran E.T., McNamee S., Lucas C., Michie J., Platt D.J., Thakker B. (2000). An outbreak of *Serratia marcescens* in two neonatal intensive care units. J. Hosp. Infect..

[46] Cilli F., Nazli-Zeka A., Arda B., Sipahi O.R., Aksit-Barik S., Kepeli N., Ozinel M.A., Gulay Z., Ulusoy S. (2018). *Serratia marcescens* sepsis outbreak caused by contaminated propofol. Am. J. Infect. Control.

[47] Blossom D., Noble-Wang J., Su J., Pur S., Chemaly R., Shams A., Jensen B., Pascoe N., Gullion J., Casey E. (2009). Multistate outbreak of *Serratia marcescens* bloodstream infections caused by contamination of prefilled heparin and isotonic sodium chloride solution syringes. Arch. Intern. Med..

[48] Sunenshine R.H., Tan E.T., Terashita D.M., Jensen B.J., Kacica M.A., Sickbert-Bennett E.E., Noble-Wang J.A., Palmieri M.J., Bopp D.J., Jernigan D.B. (2007). A multistate outbreak of *Serratia marcescens* bloodstream infection associated with contaminated intravenous magnesium sulfate from a compounding pharmacy. Clin. Infect. Dis..

[49] Civen R., Vugia D.J., Alexander R., Brunner W., Taylor S., Parris N., Wasserman R., Abbott S., Werner S.B., Rosenberg J. (2006). Outbreak of *Serratia marcescens* infections following injection of betamethasone compounded at a community pharmacy. Clin. Infect. Dis..

[50] Pan A., Dolcetti L., Barosi C., Catenazzi P., Ceruti T., Ferrari L., Magri S., Quiros Roldan E., Soavi L., Carnevale G. (2006). An outbreak of *Serratia marcescens* bloodstream infections associated with misuse of drug vials in a surgical ward. Infect. Control Hosp. Epidemiol..

[51] Tanaka T., Takahashi H., Kobayashi J.M., Ohyama T., Okabe N. (2004). A nosocomial outbreak of febrile bloodstream infection caused by heparinized-saline contaminated with *Serratia marcescens*, Tokyo, 2002. Jpn. J. Infect. Dis..

[52] Ostrowsky B.E., Whitener C., Bredenberg H.K., Carson L.A., Holt S., Hutwagner L., Arduino M.J., Jarvis W.R. (2002). *Serratia marcescens* bacteremia traced to an infused narcotic. N. Engl. J. Med..

[53] Cristina M.L., Spagnolo A.M., Orlando P., Perdelli F. (2013). The role of the environment in the spread of emerging pathogens in at-risk hospital wards. Rev. Med. Microbiol..

[54] Sehulster L., Chinn R., Arduino M.J., Carpenter J., Donlan R., Ashford D., Besser R., Fields B., McNeil M.M., Whitney C. (2004). Guidelines for in Health-Care Facilities. Recommendations from CDC and the Healthcare Infection Control Practices Advisory Committee (HICPAC).

[55] Spagnolo A.M., Sartini M., Battistella A., Casini B., Lo Pinto G., Schinca E., Cristina M.L., Hospital Infection Control Operating Group Galliera Hospital (2018). A *Clostridium difficile* outbreak in an Italian hospital: The efficacy of the multi-disciplinary and multifaceted approach. J. Prev. Med. Hyg..

[56] Spagnolo A.M., Orlando P., Panatto D., Perdelli F., Cristina M.L. (2014). An overview of carbapenem-resistant *Klebsiella pneumoniae*: Epidemiology and control measures. Rev. Med. Microbiol..

[57] Spagnolo A.M., Orlando P., Panatto D., Amicizia D., Perdelli F., Cristina M.L. (2014). *Staphylococcus aureus* with reduced susceptibility to vancomycin in healthcare settings. J. Prev. Med. Hyg..

[58] Hoyen C., Rice L., Conte S., Jacobs M.R., Walsh-Sukys M., Toltzis P. (1999). Use of real time pulsed field gel electrophoresis to guide interventions during a nursery outbreak of *Serratia marcescens* infection. Pediatr. Infect. Dis. J..

[59] Orlando P., Cristina M.L., Dallera M., Ottria G., Vitale A., Badolati G. (2008). Surface disinfection: Evaluation of the efficacy of a nebulization system spraying hydrogen peroxide. J. Prev. Med. Hyg..

[60] Perdelli F., Dallera M., Cristina M.L., Sartini M., Ottria G., Spagnolo A.M., Orlando P. (2008). A new microbiological problem in intensive care units: Environmental contamination by MRSA with reduced susceptibility to glycopeptides. Int. J. Hyg. Environ. Health.

[61] Messina G., Fattorini M., Nante N., Rosadini D., Serafini A., Tani M., Cevenini G. (2016). Time Effectiveness of Ultraviolet C Light (UVC) Emitted by Light Emitting Diodes (LEDs) in Reducing Stethoscope Contamination. Int. J. Environ. Res. Public Health.

[62] van Ogtrop M.L., van Zoeren-Grobben D., Verbakel-Salomons E.M., van Boven C.P. (1997). *Serratia marcescens* infections in neonatal departments: Description of an outbreak and review of the literature. J. Hosp. Infect..

[63] Dawczynski K., Proquitté H., Roedel J., Edel B., Pfeifer Y., Hoyer H., Dobermann H., Hagel S., Pletz M.W. (2016). Intensified colonisation screening according to the recommendations of the German Commission for Hospital Hygiene and Infectious Diseases Prevention (KRINKO): Identification and containment of a *Serratia marcescens* outbreak in the neonatal intensive care unit, Jena, Germany, 2013–2014. Infection.

